# Risk factors for possible serious bacterial infection in a rural cohort of young infants in central India

**DOI:** 10.1186/s12889-016-3688-3

**Published:** 2016-10-19

**Authors:** Marie E. Wang, Archana B. Patel, Nellie I. Hansen, Lauren Arlington, Amber Prakash, Patricia L. Hibberd

**Affiliations:** 1 Division of Infectious Diseases, Department of Medicine, Boston Children’s Hospital, Boston, MA USA; 2 Division of Global Health, Department of Pediatrics, Massachusetts General Hospital, Boston, MA USA; 3 Lata Medical Research Foundation, Nagpur, Maharashtra India; 4 RTI International, Research Triangle Park, NC USA; 5 Department of Global Health, Boston University School of Public Health, Boston University, Boston, MA USA

**Keywords:** Cohort, Possible serious bacterial infection, Young infant, India

## Abstract

**Background:**

Possible serious bacterial infection (PBSI) is a major cause of neonatal mortality worldwide. We studied risk factors for PSBI in a large rural population in central India where facility deliveries have increased as a result of a government financial assistance program.

**Methods:**

We studied 37,379 pregnant women and their singleton live born infants with birth weight ≥ 1.5 kg from 20 rural primary health centers around Nagpur, India, using data from the 2010–13 population-based Maternal and Newborn Health Registry supported by NICHD’s Global Network for Women’s and Children’s Health Research. Factors associated with PSBI were identified using multivariable Poisson regression.

**Results:**

Two thousand one hundred twenty-three infants (6 %) had PSBI. Risk factors for PSBI included nulliparity (RR 1.13, 95 % CI 1.03–1.23), parity > 2 (RR 1.30, 95 % CI 1.07–1.57) compared to parity 1–2, first antenatal care visit in the 2^nd^/3^rd^ trimester (RR 1.46, 95 % CI 1.08–1.98) compared to 1^st^ trimester, administration of antenatal corticosteroids (RR 2.04, 95 % CI 1.60–2.61), low birth weight (RR 3.10, 95 % CI 2.17–4.42), male sex (RR 1.20, 95 % CI 1.10–1.31) and lack of early initiation of breastfeeding (RR 3.87, 95 % CI 2.69–5.58).

**Conclusion:**

Infants who are low birth weight, born to mothers who present late to antenatal care or receive antenatal corticosteroids, or born to nulliparous women or those with a parity > 2, could be targeted for interventions before and after delivery to improve early recognition of signs and symptoms of PSBI and prompt referral. There also appears to be a need for a renewed focus on promoting early initiation of breastfeeding following delivery in facilities.

**Trial registration:**

This trial is registered at ClinicalTrials.gov (NCT01073475).

**Electronic supplementary material:**

The online version of this article (doi:10.1186/s12889-016-3688-3) contains supplementary material, which is available to authorized users.

## Background

Serious bacterial infection, such as meningitis, pneumonia and sepsis, is a significant cause of morbidity and mortality in neonates. Worldwide, there were an estimated 6.9 million cases in 2012 and each year serious bacterial infection is responsible for one-fifth of neonatal deaths [1, 2]. Diagnosis is challenging even in high-resource settings since signs and symptoms in neonates are often non-specific. In resource-limited settings where access to laboratory facilities is limited, diagnosis of possible serious bacterial infection (PSBI) has relied on validated clinical algorithms to identify those who would benefit from antibiotic therapy [3]. PSBI has an estimated 10 percent case fatality risk; therefore early diagnosis and treatment could save hundreds of thousands of lives [2, 4]. India bears a high burden of disease, accounting for one-quarter of all neonatal deaths, with more than 25 percent attributable to serious bacterial infections [5].

Risk factors for neonatal infection in resource-limited settings have mostly been studied in children admitted to facilities and have examined the outcome of clinical or culture-confirmed sepsis. These studies have shown that prematurity, low birth weight, prolonged rupture of membranes and maternal fever are important risk factors for neonatal sepsis, similar to resource-rich settings [6–8]. Delivery at health care facilities and skilled birth attendance has been advocated to reduce neonatal mortality, prompting the implementation of policies such as India’s conditional cash transfer scheme (Janani Suraksha Yojana) in 2005 to incentivize deliveries at facilities [9, 10]. The rates of facility delivery vary widely across India, ranging from 32–62 % in 2007–08 [11], with some areas reaching facility delivery rates greater than 90 % from 2010–2013 [12]. While facility-based delivery has the potential to improve newborn outcomes, poor infection control practices intrapartum and postpartum may contribute to the development of neonatal infections [13]. Although much has been published on PSBI in India over the last twenty years, the effect of the recent rapid change to high rates of facility delivery in India on risk factors for PSBI in the community remains less clear.

Population-based data are needed to address the full burden of PSBI, since many neonates with signs of infection, even those born in facilities, may develop signs of infection at home after discharge and do not return to a facility for evaluation. To our knowledge, there are no published population-based data on risk factors for PSBI in resource-limited settings where the overwhelming majority of births (>90 %) occur in facilities [2]. Identification of babies at highest risk for PSBI is important to further reduce neonatal mortality from PSBI. Our objective was to describe the burden of and risk factors for PSBI in infants from 0–6 weeks of age in a prospective population-based pregnancy registry in central India in the era of facility deliveries.

## Methods

### Study design and setting

We analyzed data from the Nagpur, India site of the Maternal and Newborn Health Registry (MNHR) collected between January 1, 2010 and December 31, 2013. The MNHR is a multi-country prospective, population-based observational study established by the *Eunice Kennedy Shriver* National Institute of Child Health and Human Development (NICHD) Global Network for Women's and Children's Health Research to track pregnancy and birth outcomes. The MNHR study site in Nagpur, India, covers a catchment area of 20 rural primary health centers with an estimated total population of approximately 536,000 people in 2010.

### Study procedures

Details of the MNHR study design and implementation have been published previously [14]. Briefly, registry administrators identified eligible pregnant women; 99.9 % consented to participate. Registry administrators visited participants within one week after delivery and collected information about the pregnancy, delivery and maternal and neonatal outcomes through interviews with family, birth attendants and review of available medical records. They again visited participants at six weeks postpartum and collected information about maternal and neonatal adverse conditions from delivery until six weeks of age. If the infant died, a basic death audit was conducted. Data were transmitted weekly to the data coordinating center at RTI International.

### Inclusion and exclusion criteria

We included all live born singleton infants with a birth weight of 1.5 kg and above. Babies weighing less than 1.5 kg were excluded because morbidity and mortality in this group is more likely related to their prematurity rather than just infection alone. Reported stillbirths, miscarriages and abortions were excluded. Infants were also excluded if birth weight information was missing or taken after seven days of age, or if there was no follow-up information at six weeks of age.

### Assessment of exposure variables

Information regarding maternal age and education, parity, and hemoglobin was collected at enrollment. Information on antenatal care seeking, pregnancy complications, delivery mode and location, maternal and neonatal treatment, and breastfeeding was collected at the first postpartum visit. This information was collected by medical officers from parents, birth attendants and review of available medical records. Delivery location was categorized as home/other or as referral government center (e.g. district or other government hospitals), referral private center (e.g. private clinics, nursing homes), or first level facility (public health center or sub-center) based on facility codes. Most referral government and private centers had the capability to perform C-sections, while first level facilities did not. Presence of prolonged or obstructed labor/failure to progress was indicated as a single variable in the MNHR and determined according to adapted WHO definitions. Birth weight was usually obtained on the day of birth using study scales. Low birth weight was defined as 1.5- < 2.5 kg. Initiation of breastfeeding within one hour of delivery was based on maternal report. Gestational age was not included due to concerns about the accuracy of the last reported menstrual period and lack of ultrasound availability to confirm gestational age. No data on income were collected.

Receipt of antenatal corticosteroids was identified through the MNHR and through data collected for the Antenatal Corticosteroid Trial (ACT), an 18-month cluster-randomized trial conducted by the Global Network in six countries to identify women at risk of preterm birth and promote appropriate use of antenatal corticosteroids [15]. In Nagpur, the trial was implemented in ten intervention clusters within the MNHR study area between June 11, 2012-December 11, 2013. Mothers could have also received corticosteroids outside the ACT study at their delivery facility, as antenatal corticosteroids were also available in some tertiary care centers.

### Case Definition of Possible Serious Bacterial Infection (PSBI)

We defined PSBI based on a modified version of the Young Infant Clinical Signs Study criteria [3, 16]. The presence or absence of PSBI signs and symptoms in infants 0-6 weeks of age was recorded by registry administrators retrospectively within one week of delivery and again at six weeks of age based on interviews with family, birth attendants and/or available medical records. Live born infants met the case definition for PSBI if they fulfilled one of the following criteria: 1) Presence of breathing problems/difficulty breathing, feeding problems/stopped suckling or feeding, high fever (T > 38C or estimated by touch), hypothermia (T < 35C or estimated by touch), convulsions, or bleeding/pus-like discharge from the umbilicus, 2) Cause of death coded as “infection”, or 3) Presence of free text entries under sections on symptoms or cause of death with any symptom listed in criteria 1, infection, sepsis, possible sepsis, meningitis, and/or pneumonia. There was no clinical confirmation of symptoms, information on timing of symptoms, nor did we have the ability to collect laboratory or microbiological data.

### Statistical analysis

Poisson regression models with robust variance estimation were used to assess whether maternal, peripartum and neonatal characteristics were associated with PSBI and to estimate risk ratios (RR) between groups [17, 18]. Generalized estimating equations with an independent working correlation structure and robust parameter covariance estimates were used to adjust for clustering by primary health center. We first estimated unadjusted risk ratios between PSBI and individual risk factors potentially associated with PSBI based on published literature and biological plausibility that were collected reliably in the MNHR. We then included all potential risk factors in a multivariable model for the purpose of estimating adjusted risk ratios. We included maternal, peripartum and neonatal factors, as well as year of delivery as a categorical variable to account for trends over time. Data were analyzed using STATA (version 14) [19].

We performed a sensitivity analysis to assess the relationship between the risk factors and two modified outcome variables classifying PSBI in a more restrictive manner. We first excluded those with breathing problems as a single symptom (Modified PSBI Outcome Variable 1), and then excluded those with breathing or feeding problems as a single symptom (Modified PSBI Outcome Variable 2). We did this to create a more specific outcome variable since breathing problems were present in 50 % of PSBI cases and could indicate a spectrum of symptoms, including transient respiratory distress following delivery, fast breathing (RR > 60), which is considered the least severe of the clinical signs that predict severe illness, or a more concerning sign such as severe chest indrawing [3, 20]. Feeding problems as a single symptom was excluded in Modified PSBI Outcome Variable 2 because it could also represent varying levels of difficulty, such as poor latch at birth, or a more serious sign such as inability to feed.

### Ethical approval

The study was approved by the ethics committees of Lata Medical Research Foundation, Partners Human Research Committee, and RTI International. Prior to MNHR study initiation, sensitization meetings were conducted for local community approval. Informed consent was obtained from all study participants. A data monitoring committee appointed by the *Eunice Kennedy Shriver* National Institute of Child Health and Human Development (NICHD) reviews the MNHR annually. This trial is registered at ClinicalTrials.gov (NCT01073475).

## Results

**Fig. 1:**
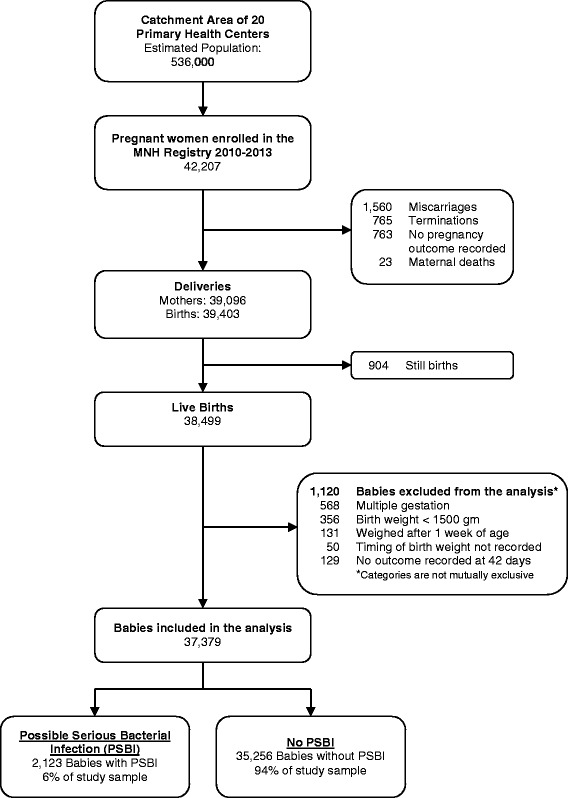
Study Profile

During the study period, we recorded 39,096 deliveries and 38,499 live births. Of the live births, 37,379 singleton infants (97 %) weighing 1.5 kg or more at birth met our inclusion criteria (Fig. 1). Of these, 2,123 (6.0 %) met the criteria for PSBI. The case fatality rate was 23 % (493/2123). Maternal, delivery, and infant characteristics are listed in Table 1.

**Table 1:** Maternal, delivery and infant characteristics^a^

	Infants with PSBI		Infants without PSBI		All infants	
	2,123	%	35,256	%	37,379	%
Year						
2010	680	32 %	8,867	25 %	9,547	26 %
2011	546	26 %	8,622	24 %	9,168	25 %
2012	450	21 %	8,684	25 %	9,134	24 %
2013	447	21 %	9,083	26 %	9,530	25 %
Maternal age						
<20	60	3 %	668	2 %	728	2 %
≥20	2,060	97 %	34,632	98 %	36,692	98 %
Maternal education						
Primary or less	414	20 %	7,153	20 %	7,567	20 %
Secondary or more	1,707	80 %	28,049	80 %	29,756	80 %
Parity						
0	1,118	53 %	16,817	48 %	17,935	48 %
1–2	935	44 %	17,520	50 %	18,455	49 %
>2	68	3 %	903	3 %	971	3 %
Maternal anemia						
No anemia (Hgb ≥11)	191	9 %	3,072	9 %	3,263	9 %
Mild anemia (Hgb 10- < 11)	877	41 %	14,786	42 %	15,663	42 %
Moderate to severe anemia (Hgb <10)	1,035	49 %	16,837	48 %	17,872	48 %
Trimester of first antenatal care visit						
First	1,304	61 %	26,855	76 %	28,159	75 %
Second or Third	453	21 %	5,814	16 %	6,267	17 %
Received antenatal care, no information on timing	364	17 %	2,566	7 %	2,930	8 %
No antenatal care	2	0.1 %	13	0.04 %	15	0.04 %
Location of delivery						
Referral government	935	44 %	15,688	44 %	16,623	44 %
Referral private	417	20 %	7,961	23 %	8,378	22 %
First level facility	656	31 %	10,276	29 %	10,932	29 %
Home/Other	110	5 %	1,304	4 %	1,414	4 %
Mode of delivery						
Vaginal	1,686	79 %	28,265	80 %	29,951	80 %
C-section^b^	437	21 %	6,991	20 %	7,428	20 %
Prolonged or obstructed labor or failure to progress						
Yes	308	15 %	4,406	12 %	4,714	13 %
No	1,812	85 %	30,803	87 %	32,615	87 %
Antenatal corticosteroids						
Yes	119	6 %	779	2 %	898	2 %
No	1,986	94 %	34,410	98 %	36,396	97 %
Birth Weight						
Normal birth weight (≥ 2500 gm)	1,325	62 %	31,009	88 %	32,334	87 %
Low birth weight (1500- < 2500 gm)	798	38 %	4,247	12 %	5,045	13 %
Initiation of breastfeeding within 1 hour after delivery						
Yes	1,470	69 %	30,767	87 %	32,237	86 %
No	634	30 %	4,346	12 %	4,980	13 %
Sex						
Male	1192	56 %	18,301	52 %	19,493	52 %
Female	931	44 %	16,950	48 %	17,881	48 %

^a^All variables had less than 2 % missing data

^b^The most common primary indications for C-section delivery were: prolonged labor/failure to progress (47 %), previous C-section (16 %), transverse/breech delivery (12 %), no clear indication (8 %), fetal distress (8 %) and severe pre-eclampsia (3 %)

### Clinical Characteristics of Infants with PSBI

Among infants with PSBI, breathing problems was the most common symptom reported (50 %), followed by fever (38 %) and feeding problems (35 %) (Table 2). Most cases (74 %) were identified by a single symptom.

**Table 2:** Reported symptoms of infants with possible serious bacterial infection

	Number	Percent
Total Number of PSBI Cases	2123	
Number of symptoms present		
No symptoms indicated^a^	26	1 %
1	1505	74 %
2	461	23 %
≥ 3	131	6 %
Breathing Problems		
Only symptom	540	25 %
With or without another symptom	1067	50 %
Feeding Problems		
Only symptom	288	14 %
With or without another symptom	737	35 %
Fever (T > 38C)		
Only symptom	598	28 %
With or without another symptom	804	38 %
Hypothermia (T < 35C)		
Only symptom	28	1 %
With or without another symptom	151	7 %
Convulsions		
Only symptom	15	1 %
With or without another symptom	48	2 %
Bleeding/pus-like discharge from the umbilicus		
Only symptom	36	2 %
With or without another symptom	48	2 %

^a^These 26 infants did not have symptoms indicated because they fulfilled the case definition of PSBI through cause of death or free text entries that did not indicate specific symptoms

### Factors Associated with PSBI

When potential risk factors were examined individually, risk of PSBI was increased for mothers who were younger than 20 years of age, and mothers who were nulliparous or had a parity > 2 (when compared to those with a parity of 1–2) (Table 3). Antenatal and delivery characteristics associated with PSBI included no antenatal care or first antenatal care visit during the second or third trimester (when compared to first trimester) and antenatal corticosteroids. Infant characteristics associated with PSBI included low birth weight, lack of early initiation of breastfeeding and male sex.

**Table 3:** Association of maternal, delivery and infant characteristics with possible serious bacterial infection

	Percent with PSBI	Unadjusted Risk Ratio	(95 % Confidence Interval)	Adjusted Risk Ratio^a^	(95 % Confidence Interval)
Year					
2010	7 %	1.00		1.00	
2011	6 %	0.84	(0.60, 1.17)	0.81	(0.57, 1.15)
2012	5 %	0.69	(0.48, 1.00)	0.66	(0.46, 0.93)
2013	5 %	0.66	(0.42, 1.04)	0.66	(0.36, 1.20)
Maternal age					
<20	8 %	1.46	(1.21, 1.70)	1.11	(0.93, 1.32)
≥ 20	6 %	1.00		1.00	
Maternal education					
Primary or less	5 %	0.95	(0.87, 1.05)	0.89	(0.80, 1.00)
Secondary or more	5 %	1.00		1.00	
Parity					
0	6 %	1.23	(1.11, 1.36)	1.13	(1.03, 1.23)
1–2	5 %	1.00		1.00	
>2	7 %	1.38	(1.15, 1.66)	1.30	(1.07, 1.57)
Maternal anemia					
No anemia (Hgb ≥11)	6 %	1.00		1.00	
Mild anemia (Hgb 10- < 11)	6 %	0.96	(0.71, 1.30)	0.96	(0.79, 1.16)
Moderate to severe anemia (Hgb <10)	6 %	0.99	(0.58, 1.70)	0.92	(0.64, 1.31)
Trimester of first antenatal care visit					
First	5 %	1.00		1.00	
Second or Third	7 %	1.56	(1.12, 2.17)	1.46	(1.08, 1.98)
Received antenatal care, no information on timing	12 %	2.68	(1.65, 4.36)	2.27	(1.29, 4.00)
No antenatal care	13 %	2.88	(1.40, 5.93)	3.21	(1.66, 6.20)
Location of delivery					
Referral government	6 %	1.00		1.00	
Referral private	5 %	0.89	(0.67, 1.17)	0.83	(0.65, 1.06)
First level facility	6 %	1.07	(0.88, 1.30)	1.14	(1.00, 1.30)
Home/Other	8 %	1.38	(1.04, 1.84)	1.16	(0.86, 1.57)
Mode of delivery					
Vaginal	6 %	1.00		1.00	
C-section	6 %	1.04	(0.88, 1.25)	0.48	(0.36, 0.63)
Prolonged or obstructed labor or failure to progress					
Yes	7 %	1.18	(0.84, 1.64)	1.22	(0.87, 1.69)
No	6 %	1.00		1.00	
Antenatal corticosteroids					
Yes	13 %	2.43	(1.86, 3.16)	2.04	(1.60, 2.61)
No	5 %	1.00		1.00	
Birth Weight					
Normal birth weight (≥ 2500 gm)	4 %	1.00		1.00	
Low birth weight (1500- < 2500 gm)	16 %	3.86	(2.68, 5.55)	3.10	(2.17, 4.42)
Initiation of breastfeeding within 1 hour after delivery					
Yes	5 %	1.00		1.00	
No	13 %	2.79	(1.95, 3.98)	3.87	(2.69, 5.58)
Sex					
Male	6 %	1.17	(1.07, 1.29)	1.20	(1.10, 1.31)
Female	5 %	1.00		1.00	

^a^36,410 of 37,379 (97 %) observations were included in the multivariable model

On multivariable analysis, low birth weight (RR 3.10, 95 % confidence interval [CI] 2.17–4.42) and lack of early initiation of breastfeeding (RR 3.87, 95 % CI 2.69–5.58) were highly associated with risk of PSBI. Other characteristics associated with a significantly increased risk of PSBI included: nulliparity (RR 1.13, 95 % CI 1.03–1.23), parity > 2 (RR 1.30, 95 % CI 1.07–1.57), first antenatal care visit in the second or third trimester (RR 1.46, 95 % CI 1.08–1.98), no antenatal care (RR 3.21, 95 % CI 1.66–6.20), antenatal corticosteroids (RR 2.04, 95 % CI 1.60–2.61), and male sex (RR 1.20, 95 % CI 1.10–1.31). C-section delivery was associated with a lower risk of PSBI (RR 0.48, 95 % CI 0.36–0.63).

Because we found an unexpected association between C-section delivery and PSBI in the multivariate analysis that was not present in the univariate analysis, we assessed whether each of the other variables in the model could have modified the effect of delivery mode on PSBI. We found evidence of effect modification by early initiation of breastfeeding. We assessed these associations further by including a 4-category variable that combined delivery mode and early initiation of breastfeeding (vaginal delivery/breastfeeding within 1 h, vaginal delivery/no early breastfeeding, C-section delivery/breastfeeding within 1 h, C-section delivery/no early breastfeeding) in a second multivariable model that also included all the other variables included in the primary model. The rate of early initiation of breastfeeding following vaginal delivery was 96 %, compared to 49 % for C-sections (Table 4). When compared to babies delivered by vaginal delivery who initiated breastfeeding within one hour after delivery, the risk of PSBI was not significantly different for babies delivered by C-section who had early initiation of breastfeeding (RR 0.78, 95 % CI 0.58–1.04). However, the risk of PSBI was significantly higher among babies delivered by C-section who did not have early initiation of breastfeeding (RR 1.66, 95 % CI 1.30–2.11) and highest among babies delivered by vaginal delivery who did not have early initiation of breastfeeding (RR 4.74, 95 % CI 3.13–7.17). The sub-group of infants who were born by vaginal delivery and were not breastfed early had a higher percentage of low birth weight infants (375/1202, 31 %) and a higher case fatality rate (195/1202, 16.2 %) when compared with the three other sub-groups. Infants born by vaginal delivery who were breastfed early had 13 % low birth weight infants (3,640/28,642) with a case fatality rate of 1.2 % (333 deaths); infants born by C-section who were breastfed early had 13 % low birth weight infants (459/3595) and a case fatality rate of 1.3 % (48 deaths); and those delivered by C-section who were not breastfed early had 14 % low birth weight infants (541/3778) and a case fatality rate of 1.9 % (72 deaths).

**Table 4:** Interaction between delivery mode and early initiation of breastfeeding with outcome of possible serious bacterial infection

	Number (%) with PSBI	Total Number of Infants (%)	Adjusted Risk Ratio^a^	95 % CI
Vaginal Delivery				
Early Initiation of Breastfeeding	1346 (4)	28,642 (96)	1.00	
Lack of early initiation of breastfeeding	330 (27)	1,202 (4)	4.74	(3.13–7.17)
C-section				
Early Initiation of Breastfeeding	124 (3)	3,595 (49)	0.78	(0.58–1.04)
Lack of early initiation of breastfeeding	304 (8)	3,778 (51)	1.66	(1.30–2.11)

^a^Multivariable regression model adjusted for year, age, parity, maternal education, timing of first antenatal care visit, maternal anemia, location of delivery, prolonged labor, infant sex, birth weight, and the 4-level variable listed above combining delivery mode and breastfeeding status

### Sensitivity analysis

After exclusion of cases based on breathing problems as a single symptom, the characteristics significantly associated with PSBI remained the same (Additional file 1: Table S1). Results were also similar after exclusion of cases based on either breathing or feeding problems as a single symptom with the exception that the risk of PSBI for parity > 2 compared to 1–2 was no longer significantly different.

## Discussion

Our results show that PSBI occurred in 6 % of babies born to a population-based cohort of 37,379 pregnant women in rural central India, where 96 % of deliveries occurred in facilities. This is similar to the 5.1 % rate of PSBI found in a large community-based study in Ghana where 68 % of deliveries occurred in a facility [21]. It is also consistent with the pooled estimate of PSBI incidence in South Asia (7.2 %, 95 % CI 5.0–9.3) developed by Seale et al. using data from multiple studies [2]. However our estimate was lower than the 14.4 % and 9.2 % rates found in community-based studies in Bangladesh [22] and Nepal [23], respectively, where the majority of deliveries (>80 %) occurred at home.

In this population-based study with a majority of facility deliveries, many of the factors associated with PSBI were similar to those previously reported in babies admitted to facilities [6–8]. Factors that were associated with an increased risk of PSBI in our population included low birth weight, nulliparity, parity > 2, first antenatal care visit after the first trimester or no antenatal care, lack of early initiation of breastfeeding, antenatal corticosteroids, and male sex.

Within low-resource settings, low birth weight is an established risk factor for neonatal mortality and has been shown to be a risk factor for neonatal sepsis in facility-based studies [1, 6–8, 24]. Nulliparity has also been shown to be associated with early onset sepsis in a large South African cohort (RR 1.8; 95 % CI 1.4–2.3) [7]. In that cohort, nulliparity was associated with a number of traditional sepsis risk factors, such as prolonged labor and greater number of vaginal examinations. Appropriate antenatal care has been associated with improved neonatal outcomes [9]. A facility-based study in Uganda found that no antenatal care was associated with a 3-times higher odds of culture-confirmed sepsis compared to mothers who did receive antenatal care (odds ratio 3.21, 95 % CI 1.24–8.33) [25].

The finding of a lower rate of PSBI among C-section deliveries in the multivariable model was unexpected. Differences in reasons for not breastfeeding early may have contributed to this finding. In the babies who did receive early breastfeeding, risk of PSBI did not differ by delivery type. In the babies who were not breastfed early, the risk of PSBI was higher in vaginal deliveries compared to C-sections. This suggests that the difference in PSBI rates seen between vaginal and C-section deliveries may have been driven by the population of babies that was not breastfed early and their differences in baseline risk. The small percentage of babies delivered vaginally who were not breastfed early (4 %) may have been sicker, requiring medical attention that prevented breastfeeding immediately after birth. In contrast, 51 % of babies delivered by C-section did not have early breastfeeding, which was more likely due to maternal post-operative recovery rather than an indicator of the health of the baby. Nevertheless, babies delivered by C-section who were not breastfed early had a higher risk of PSBI compared to babies delivered by C-section who were. Breastfeeding has been associated with decreased rates of neonatal sepsis and other neonatal infections [26, 27]. One meta-analysis found that initiation of breastfeeding within 24 h of birth was associated with a lower neonatal mortality rate (RR 0.55, 95 % CI 0.36–0.84) [28]. As more births are occurring in facilities and the percentage of C-sections are rising [29], improving rates of early initiation of breastfeeding following C-section is a potentially important area for intervention.

Our finding of the association between antenatal corticosteroids and PSBI deserves further exploration. Antenatal corticosteroids have been shown to reduce neonatal morbidity and mortality from pre-term birth in hospitals [30]. However, a recent cluster-randomized trial of antenatal corticosteroid use in six low- and middle-income countries showed an increased 28-day neonatal mortality rate in the group who received corticosteroids (RR 1.12, 95 % CI 1.02–1.22) [15]. This may have been due to the administration of corticosteroids to women thought to be at risk for preterm birth who ultimately delivered late preterm or term infants. On secondary analysis of these data, there was no significant increase in risk of PSBI in the intervention clusters who received antenatal corticosteroids when compared to the control clusters [31]. When the composite outcome of PSBI and death was analyzed, risk was increased in the intervention clusters (RR 1.17, 95 % CI 1.04–1.30), which was driven by the difference among infants with a birth weight greater than or equal to the 25^th^ percentile. Within our study population of babies with birth weight ≥ 1.5 kg, corticosteroids may have resulted in a compromised immune system and increased risk for infection in the neonate if the corticosteroids were administered to the mother after week 34 of pregnancy. It is also possible that our PSBI outcome variable misclassified cases because it was based only on reported symptoms. Therefore, further study of antenatal corticosteroid use and its relationship with neonatal infection as assessed by clinical and laboratory criteria is urgently required as antenatal corticosteroids are being scaled up in low-resource settings [32].

This study has important strengths. We determined rates of PSBI up to 42 days of age in a large, population-based cohort of mothers and babies over a four-year period. Trained study staff prospectively collected information on PSBI, risk factors and outcomes, reducing the risk of selection bias when babies admitted to facilities with PSBI are studied. Our study also has several limitations. First, PSBI was defined on the basis of clinical signs and symptoms, not laboratory data or microbiological confirmation of infection. However, because laboratory diagnosis of infection in young infants is difficult in resource-limited settings, identification of PSBI has traditionally relied on versions of the definition used in this study [2]. Secondly, we did not have the ability to determine the exact timing of PSBI symptoms, nor did we require clinical confirmation of the symptoms. Questions regarding the symptoms of breathing or feeding problems, which are the least objective measures, may have been interpreted differently by respondents and/or study staff. We addressed this issue by excluding the babies with breathing or feeding problems as a single symptom in our sensitivity analysis and obtained results similar to those from the primary analysis. Third, the MNHR was not specifically designed to assess rates of PSBI and therefore does not include all factors that may be associated with PSBI, such as the duration of rupture of membranes, maternal fever, etc. We also did not include gestational age in our analysis and are thus unable to separate the effect of birth weight from that of prematurity. We chose not to include estimates of gestational age based on last menstrual period dating due to the poor reliability of this dating in our population and the inability to confirm dates with ultrasound. Lastly, because the study was completed at a single site in India, which consists of many diverse states, the findings may not be representative of the entire country.

## Conclusions

Since neonatal infections are the third most common cause of neonatal deaths worldwide and the leading cause of death in the late neonatal period (7–27 days) [1], achieving significant reductions in neonatal mortality requires improvements in the identification and treatment of neonatal infections. Our study in a large rural population-based cohort around Nagpur, India, with a high rate of facility delivery, showed that many of the known risk factors for PSBI such as low birth weight and late or no antenatal care, were still present. Our data suggest that infants born to nulliparous women, women who present late to antenatal care, those receiving antenatal corticosteroids and infants who are low birth weight could be targeted for extra antenatal and post-natal visits for early recognition of signs and symptoms of PSBI and prompt referral. Promoting early breastfeeding following C-section may also be a potential opportunity for intervention.

## Additional file

Additional file 1: Table S1. Sensitivity Analyses for Multivariable Model with Outcome of Possible Serious Bacterial Infection. (DOC 77 kb)

## Abbreviations

ACT: Antenatal corticosteroid trial
CI: Confidence interval
MNHR: Maternal and newborn health registry
NICHD: *Eunice Kennedy Shriver* National Institute of Child Health and Human Development
PSBI: Possible serious bacterial infection
RR: Risk ratio
WHO: World Health Organization

## References

[1] Lawn JE, Blencowe H, Oza S, You D, Lee AC, Waiswa P (2014). Every Newborn: progress, priorities, and potential beyond survival. Lancet.

[2] Seale AC, Blencowe H, Manu AA, Nair H, Bahl R, Qazi SA (2014). Estimates of possible severe bacterial infection in neonates in sub-Saharan Africa, south Asia, and Latin America for 2012: a systematic review and meta-analysis. Lancet Infect Dis.

[3] Clinical signs that predict severe illness in children under age 2 months: a multicentre study. Lancet. 2008;371(9607):135-42. doi:10.1016/s0140-6736(08)60106-3 10.1016/S0140-6736(08)60106-3 18191685

[4] Zaidi AK, Ganatra HA, Syed S, Cousens S, Lee AC, Black R (2011). Effect of case management on neonatal mortality due to sepsis and pneumonia. BMC Public Health.

[5] Bassani DG, Kumar R, Awasthi S, Morris SK, Paul VK, Shet A (2010). Causes of neonatal and child mortality in India: a nationally representative mortality survey. Lancet.

[6] Leal YA, Álvarez-Nemegyei J, Velázquez JR, Rosado-Quiab U, Diego-Rodríguez N, Paz-Baeza E (2012). Risk factors and prognosis for neonatal sepsis in southeastern Mexico: analysis of a four-year historic cohort follow-up. BMC Pregnancy Childbirth.

[7] Schrag SJ, Cutland CL, Zell ER, Kuwanda L, Buchmann EJ, Velaphi SC (2012). Risk factors for neonatal sepsis and perinatal death among infants enrolled in the prevention of perinatal sepsis trial, Soweto, South Africa. Pediatr Infect Dis J.

[8] Kayange N, Kamugisha E, Mwizamholya DL, Jeremiah S, Mshana SE (2010). Predictors of positive blood culture and deaths among neonates with suspected neonatal sepsis in a tertiary hospital, Mwanza-Tanzania. BMC Pediatr.

[9] Bhutta ZA, Das JK, Bahl R, Lawn JE, Salam RA, Paul VK (2014). Can available interventions end preventable deaths in mothers, newborn babies, and stillbirths, and at what cost?. Lancet.

[10] Lim SS, Dandona L, Hoisington JA, James SL, Hogan MC, Gakidou E (2010). India's Janani Suraksha Yojana, a conditional cash transfer programme to increase births in health facilities: an impact evaluation. Lancet.

[11] Patel R, Ladusingh L (2015). Do Physical Proximity and Availability of Adequate Infrastructure at Public Health Facility Increase Institutional Delivery? A Three Level Hierarchical Model Approach. PLoS One.

[12] Goudar SS, Goco N, Somannavar MS, Vernekar SS, Mallapur AA, Moore JL (2015). Institutional deliveries and perinatal and neonatal mortality in Southern and Central India. Reprod Health.

[13] Zaidi AK, Huskins WC, Thaver D, Bhutta ZA, Abbas Z, Goldmann DA (2005). Hospital-acquired neonatal infections in developing countries. Lancet.

[14] Bose CL, Bauserman M, Goldenberg RL, Goudar SS, McClure EM, Pasha O (2015). The Global Network Maternal Newborn Health Registry: a multi-national, community-based registry of pregnancy outcomes. Reprod Health.

[15] Althabe F, Belizan JM, McClure EM, Hemingway-Foday J, Berrueta M, Mazzoni A (2015). A population-based, multifaceted strategy to implement antenatal corticosteroid treatment versus standard care for the reduction of neonatal mortality due to preterm birth in low-income and middle-income countries: the ACT cluster-randomised trial. Lancet.

[16] Hibberd PL, Hansen NI, Wang ME, Goudar SS, Pasha O, Esamai F (2016). Trends in the incidence of possible severe bacterial infection and case fatality rates in rural communities in Sub-Saharan Africa, South Asia and Latin America, 2010-2013: a multicenter prospective cohort study. Reprod Health.

[17] Zou GY, Donner A (2013). Extension of the modified Poisson regression model to prospective studies with correlated binary data. Stat Methods Med Res.

[18] Yelland LN, Salter AB, Ryan P (2011). Performance of the modified Poisson regression approach for estimating relative risks from clustered prospective data. Am J Epidemiol.

[19] StataCorp (2015). Stata Statistical Software: Release 14.

[20] Zaidi AK, Baqui AH, Qazi SA, Bahl R, Saha S, Ayede AI (2013). Scientific rationale for study design of community-based simplified antibiotic therapy trials in newborns and young infants with clinically diagnosed severe infections or fast breathing in South Asia and sub-Saharan Africa. Pediatr Infect Dis J.

[21] Kirkwood BR, Manu A, ten Asbroek AH, Soremekun S, Weobong B, Gyan T (2013). Effect of the Newhints home-visits intervention on neonatal mortality rate and care practices in Ghana: a cluster randomised controlled trial. Lancet.

[22] Arifeen SE, Mullany LC, Shah R, Mannan I, Rahman SM, Talukder MR (2012). The effect of cord cleansing with chlorhexidine on neonatal mortality in rural Bangladesh: a community-based, cluster-randomised trial. Lancet.

[23] Khanal S, Sharma J, Gc VS, Dawson P, Houston R, Khadka N (2011). Community health workers can identify and manage possible infections in neonates and young infants: MINI--a model from Nepal. J Health Popul Nutr.

[24] Dhaded SM, Somannavar MS, Vernekar SS, Goudar SS, Mwenche M, Derman R (2015). Neonatal mortality and coverage of essential newborn interventions 2010 - 2013: a prospective, population-based study from low-middle income countries. Reprod Health.

[25] John B, David M, Mathias L, Elizabeth N (2015). Risk factors and practices contributing to newborn sepsis in a rural district of Eastern Uganda, August 2013: a cross sectional study. BMC Res Notes.

[26] Bhutta ZA, Yusuf K (1997). Early-onset neonatal sepsis in Pakistan: a case control study of risk factors in a birth cohort. Am J Perinatol.

[27] Ashraf RN, Jalil F, Zaman S, Karlberg J, Khan SR, Lindblad BS (1991). Breast feeding and protection against neonatal sepsis in a high risk population. Arch Dis Child.

[28] Debes AK, Kohli A, Walker N, Edmond K, Mullany LC (2013). Time to initiation of breastfeeding and neonatal mortality and morbidity: a systematic review. BMC Public Health.

[29] Vogel JP, Betran AP, Vindevoghel N, Souza JP, Torloni MR, Zhang J (2015). Use of the Robson classification to assess caesarean section trends in 21 countries: a secondary analysis of two WHO multicountry surveys. Lancet Global Health.

[30] Roberts D, Dalziel S. Antenatal corticosteroids for accelerating fetal lung maturation for women at risk of preterm birth. Cochrane Database Syst Rev. 2006(3):Cd004454. doi:10.1002/14651858.CD004454.pub2 10.1002/14651858.CD004454.pub2 16856047

[31] Althabe F, Thorsten V, Klein K, McClure EM, Hibberd PL, Goldenberg RL (2016). The Antenatal Corticosteroids Trial (ACT)'s explanations for neonatal mortality - a secondary analysis. Reprod Health.

[32] World Health Organization. WHO recommendations on interventions to improve preterm birth outcomes. Geneva: World Health Organization; 2015. 26447264

