# Supplementary material for: Risk factors for possible serious bacterial infection in a rural cohort of young infants in central India
Source: BMC Public Health. 2016 Oct 19;16:1097. doi: 10.1186/s12889-016-3688-3 (PMC5070173; doi:10.1186/s12889-016-3688-3)
Supplement: Additional file 1: Table S1. — Sensitivity Analyses for Multivariable Model with Outcome of Possible Serious Bacterial Infection. (DOC 77 kb) [file 12889_2016_3688_MOESM1_ESM.doc]

Additional file 1: Table S1. Sensitivity Analyses for Multivariable Model with Outcome of Possible Serious Bacterial Infection

|  | Outcome: Modified  PSBI Variable 1^a^ | | Outcome: Modified  PSBI Variable 2^b^ | |
| --- | --- | --- | --- | --- |
|  | (number with outcome = 1583) | | (number with outcome=1295) | |
|  | Adjusted Risk Ratio | (95% Confidence Interval) | Adjusted Risk Ratio | (95% Confidence Interval) |
| **Year** |  |  |  |  |
| 2010 | 1.00 |  | 1.00 |  |
| 2011 | 0.87 | (0.63, 1.20) | 0.86 | (0.60, 1.24) |
| 2012 | 0.72 | (0.51, 1.01) | 0.62 | (0.39, 0.98) |
| 2013 | 0.75 | (0.40, 1.38) | 0.75 | (0.36, 1.55) |
| **Maternal age** |  |  |  |  |
| <20 | 1.12 | (0.87, 1.44) | 1.17 | (0.87, 1.57) |
| ≥ 20 | 1.00 |  | 1.00 |  |
| **Maternal education** |  |  |  |  |
| Primary or less | 0.92 | (0.87, 1.44) | 0.93 | (0.78, 1.11) |
| Secondary or more | 1.00 |  | 1.00 |  |
| **Parity** |  |  |  |  |
| 0 | 1.19 | (1.07, 1.32) | 1.17 | (1.07, 1.27) |
| 1-2 | 1.00 |  | 1.00 |  |
| >2 | 1.31 | (1.05, 1.63) | 1.24 | (0.95, 1.61) |
| **Maternal anemia** |  |  |  |  |
| No anemia (Hgb ≥11) | 1.00 |  | 1.00 |  |
| Mild anemia (Hgb 10-<11) | 0.94 | (0.74, 1.18) | 0.89 | (0.72, 1.11) |
| Moderate to severe anemia (Hgb <10) | 0.87 | (0.56, 1.36) | 0.79 | (0.50, 1.27) |
| **Trimester of first antenatal care visit** |  |  |  |  |
| First | 1.00 |  | 1.00 |  |
| Second or Third | 1.60 | (1.12, 2.29) | 1.72 | (1.16, 2.53) |
| Received antenatal care, no information on timing | 2.45 | (1.18, 5.09) | 2.81 | (1.21, 6.57) |
| No antenatal care | 4.23 | (2.19, 8.16) | 5.10 | (2.76, 9.42) |
| **Location of delivery** |  |  |  |  |
| Referral government | 1.00 |  | 1.00 |  |
| Referral private | 0.78 | (0.57, 1.06) | 0.71 | (0.50, 1.02) |
| First level facility | 1.13 | (0.99, 1.30) | 1.05 | (0.92, 1.21) |
| Home/Other | 1.23 | (0.88, 1.71) | 1.16 | (0.82, 1.65) |
| **Mode of delivery** |  |  |  |  |
| Vaginal | 1.00 |  | 1.00 |  |
| C-section | 0.47 | (0.33, 0.66) | 0.45 | (0.29, 0.70) |
| **Prolonged or obstructed labor or failure to progress** |  |  |  |  |
| Yes | 0.98 | (0.78, 1.22) | 1.00 | (0.79, 1.26) |
| No | 1.00 |  | 1.00 |  |
| **Antenatal corticosteroids** |  |  |  |  |
| Yes | 2.09 | (1.58, 2.77) | 2.21 | (1.60, 3.06) |
| No | 1.00 |  | 1.00 |  |
| **Birth Weight** |  |  |  |  |
| Normal birth weight (≥ 2500 gm) | 1.00 |  | 1.00 |  |
| Low birth weight (1500-<2500 gm) | 3.26 | (2.24, 4.74) | 2.83 | (1.90, 4.19) |
| **Initiation of breastfeeding within 1 hour after delivery** |  |  |  |  |
| Yes | 1.00 |  | 1.00 |  |
| No | 3.87 | (2.52, 5.95) | 3.95 | (2.40, 6.52) |
| **Sex** |  |  |  |  |
| Male | 1.24 | (1.13, 1.37) |  | 1.20 |
| Female | 1.00 |  |  | 1.00 |

^a^ Outcome variable of PSBI excluding infants with breathing problems as a single symptom

^b^ Outcome variable of PSBI excluding infants with breathing or feeding problems as a single symptom
